# Effect of distant-image screen technology (DIST) on delaying myopia onset in pre-myopia children: study protocol for a1-year randomized controlled trial

**DOI:** 10.1186/s13063-026-09696-2

**Published:** 2026-04-11

**Authors:** Liying Zou, Xiaoru Li, Jiawei Zhou, Meiping Xu

**Affiliations:** 1 School of Ophthalmology & Optometry and Eye Hospital, Wenzhou Medical University, Wenzhou, Zhejiang China; 2 National Clinical Research Center for Ocular Disease, Wenzhou, Zhejiang China

**Keywords:** Myopia prevention, Pre-myopia, Distant-image screen technology, Progression, Axial length

## Abstract

**Background:**

Myopia is an escalating global health issue, particularly among adolescents, and its increasing prevalence is associated with a rising burden of ocular complications that adversely affect quality of life and strain healthcare resources. Extensive evidence links prolonged near work to myopia progression, prompting the development of innovative control strategies. One promising approach is the distant-image screen (DIST), which transforms a nearby real image into a virtual one that appears much farther away, thereby reducing the accommodative stress typically induced by prolonged near work. This study is designed to evaluate the efficacy of DIST in delaying the onset of myopia among pre-myopic children.

**Methods:**

This is a 1-year, multi-arm randomized controlled trial involving 192 children, who will be randomly assigned in a 1:1:1 ratio to one of three groups: (1) a DIST group; (2) a Combined Intervention group, which will receive both DIST and an optical defocusing intervention; and (3) a control group, engaging in regular near work without the use of DIST. The primary objective is to assess whether the use of DIST—alone or in combination with optical defocusing—can effectively delay the onset of myopia in pre-myopic children. The primary outcome is the proportion of myopia onset, and the secondary outcomes are the proportion of fast myopia progressors, change in spherical equivalent progression, and change in axial length at each follow-up point.

**Discussion:**

The study aims to determine the independent efficacy of DIST as well as its potential synergistic benefits when combined with optical defocusing techniques. In the context of increasing academic demands and near work exposure, DIST offers a space-efficient, practical solution that could alleviate visual strain without interfering with learning. By providing robust data on both refractive and ocular structural changes, the findings may inform personalized myopia prevention strategies. If successful, DIST could serve as a valuable adjunct to current myopia control methods, ultimately reducing the public health burden of myopia.

**Trial registration:**

Chinese Clinical Trial Registry (ChiCTR), ChiCTR2400082078. Registered on 20 March 2024. https://www.chictr.org.cn/showproj.html?proj=221835.

## Administrative information

Note: the numbers in curly brackets in this protocol refer to SPIRIT checklist item numbers. The order of the items has been modified to group similar items (see http://www.equator-network.org/reporting-guidelines/spirit-2013-statement-defining-standard-protocol-items-for-clinical-trials/).

**Table:** 

Title {1}	Effect of distant-image screen technology (DIST) on delaying myopia onset in pre-myopia children: study protocol for a 1-year randomized controlled trial
Trial registration {2a and 2b}	Chinese Clinical Trial Registry (ChiCTR), ChiCTR2400082078. Registered on 20 March 2024. https://www.chictr.org.cn/showproj.html?proj=221835
Protocol version {3}	Protocol version 1. Protocol date: April 21, 2025 Protocol version 2. Protocol date: March 30, 2026
Funding {4}	This investigator-initiated trial is sponsored by the Eye Hospital of Wenzhou Medical University, Zhejiang, China, which will provide all necessary medical instruments, examinations, and evaluation services free of charge. No external funding is involved, ensuring the study’s independence and impartiality.
Author details {5a}	Liying Zou^1,2^, Xiaoru Li^1,2^, Jiawei Zhou^1,2^ and Meiping Xu^1,2*^ ^1^School of Ophthalmology & Optometry and Eye Hospital, Wenzhou Medical University, Wenzhou, Zhejiang, China ^2^National Clinical Research Center for Ocular Disease, Wenzhou, Zhejiang, China *Corresponding author
Name and contact information for the trial sponsor {5b}	Eye Hospital of Wenzhou Medical University No. 270, College West Road, Wenzhou, Zhejiang, China Telephone: 0577–88068888 Email: eyehospital@mail.eye.ac.cn
Role of sponsor {5c}	The sponsor of this trial, Eye Hospital of Wenzhou Medical University, assumes overall responsibility for the initiation, management, and funding of the study. Specific roles and responsibilities include ethical and regulatory compliance, provision of resources, trial oversight, data management and integrity, independence of analysis.

## Introduction

### Background and rationale {6a}

Myopia is a prevalent refractive error that significantly contributes to visual impairment worldwide. Over the past few decades, the global prevalence of myopia has risen sharply, with epidemiological studies predicting that by 2050, nearly half of the world’s population (approximately 4.758 billion individuals) will be myopic, including almost 1 billion affected by high myopia [1]. The high prevalence of myopia, especially among adolescents in certain populations (80–90%), has reached alarming levels [2, 3]. Myopia has raised considerable public health concerns due to its associated risk of severe ocular complications, such as cataracts, glaucoma, and retinal detachment [4–7]. These complications not only impair vision but also impose a substantial burden on patients’ quality of life and healthcare systems [8, 9]. Moreover, myopia can adversely impact children’s and adolescents’ learning and daily lives, potentially leading to reduced self-esteem and social difficulties [10–12]. Consequently, the prevention and control of myopia have emerged as pressing global public health priorities.

A growing body of evidence highlights the association between prolonged near work—such as reading or engaging in close-up tasks—and the onset and progression of myopia in children and adolescents [13–17]. Mechanistically, near work has been linked to alterations in choroidal thickness, a biomarker increasingly studied in the context of myopia development [18], suggesting that reducing near work exposure could mitigate myopia progression [19]. Furthermore, the extended duration of near work has been shown to have an impact. This understanding has driven the innovation of novel technologies aimed at addressing the challenges of near work.

The distant-image screen technology (DIST) is a novel myopia prevention and control approach developed in the past 2 years. DIST utilizes the “Birdbath” optical design principle and freeform mirror technology to project near scenes to a perceived distance beyond 3 m. This innovation theoretically reduces the visual strain associated with near work while allowing children and adolescents to engage in tasks such as reading and note-taking. A previous clinical study has confirmed that using the DIST for reading does not compromise reading efficiency or increase visual fatigue, providing early support for its feasibility and safety in daily use [20].

In parallel, optical defocus has been widely recognized as an effective strategy for myopia control, with clinical applications such as peripheral defocus glasses and contact lenses [21–24]. While the potential synergistic effects of DIST and optical defocus technologies remain unexplored, their combination may represent a promising avenue for enhancing myopia control outcomes. This research aims to investigate whether DIST and integrating DIST with optical defocus principles can further optimize myopia prevention strategies, offering new insights into evidence-based myopia management.

### Objectives {7}

Based on the aforementioned theories, our primary objective was to evaluate the efficacy of DIST and integrating DIST with optical defocus principles on delaying the onset of myopia, specifically in postponing the initial diagnosis of myopia in pre-myopic children. Additionally, we aimed to determine its impact on mitigating myopic refractive shift and slowing ocular axis elongation. We hypothesized that the DIST intervention would demonstrate superior efficacy in these outcomes compared to the control group, highlighting its potential as a targeted strategy for myopia prevention.

### Trial design {8}

**Fig. 1:**
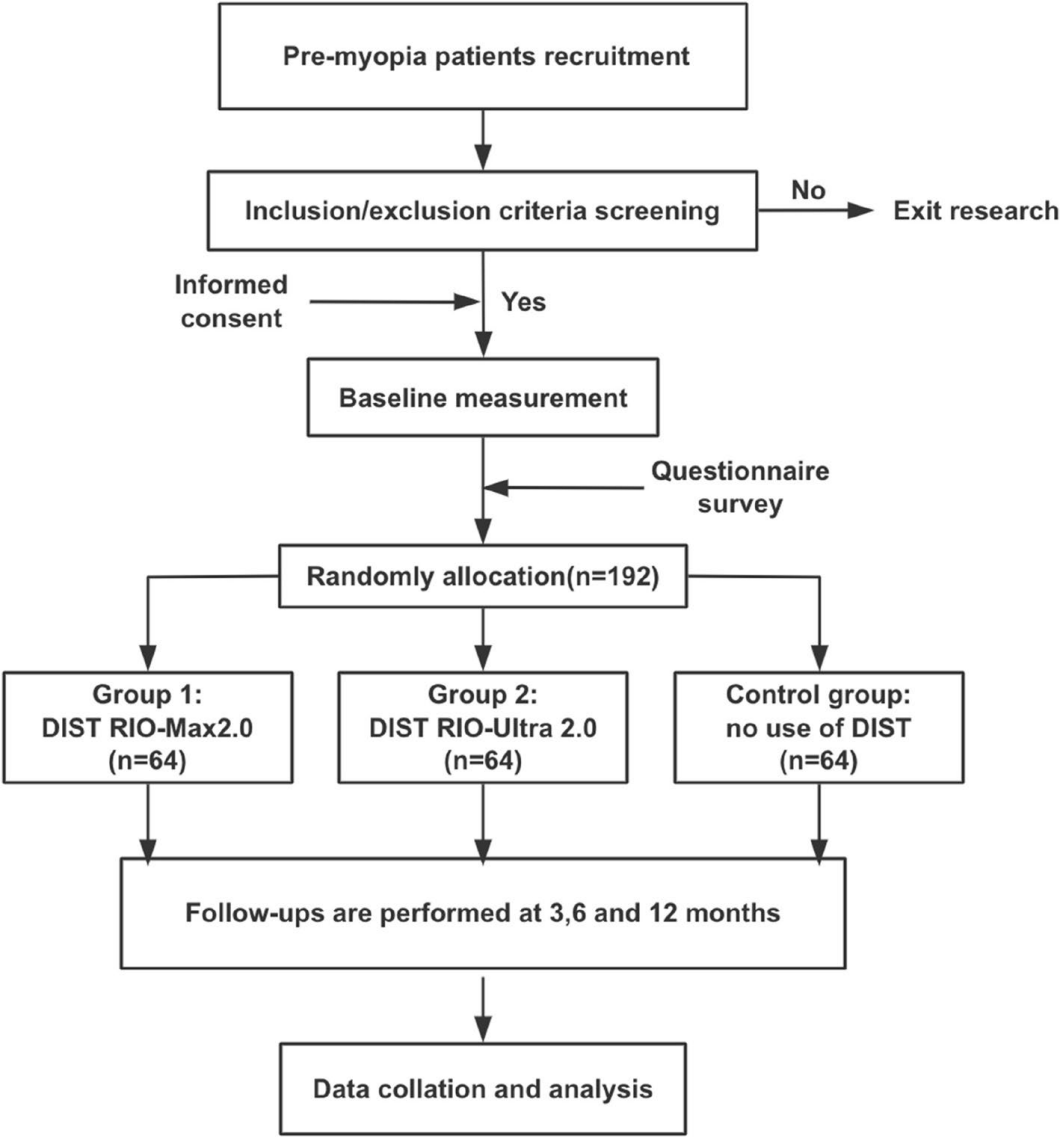
The flowchart of the study

This study will be a randomized, superiority-controlled trial with a parallel-group design. One hundred ninety-two pre-myopic children will be randomly divided into three groups of 64 subjects in a 1:1:1 ratio. The three groups are no use of DIST, DIST alone (RIO-Max 2.0), and DIST optimized by combining the optical defocusing technique (RIO-Ultra 2.0). The complete study flowchart is shown in the figure (Fig. 1). The protocol and all study procedures are approved by the Medical Ethics Committee of the Affiliated Eye Hospital of Wenzhou Medical University (2024–016-K-015–05) and conducted following the ethical standards of the Declaration of Helsinki. The protocol has been registered with the Chinese Clinical Trial Registry (ChiCTR2400082078) and uses the SPIRIT reporting guidelines [25]. Subjects are required to sign an ethical informed consent form before participating in the experiment.

## Methods: participants, interventions, and outcomes

### Study setting {9}

Participants in this study will be recruited from the Eye Hospital of Wenzhou Medical University. Screening and baseline examinations will be conducted in the outpatient clinic of the Eye Hospital, using advanced and comprehensive equipment to ensure accurate and complete data collection. Participants assigned to the two intervention groups using DIST will take the devices home. Follow-up supervision will be conducted by the researchers to ensure adherence to the study protocol and proper implementation of the intervention.

### Eligibility criteria {10}

All subjects will undergo initial screening by ophthalmologists and investigators before enrollment. This screening will include inquiries about general health condition, family and disease history, allergies, and current medications. Potential subjects who meet these preliminary criteria will then be further assessed for ocular health and lifestyle habits based on the established inclusion and exclusion criteria.

Inclusion criteria are as follows:

1. Age: 6–10 years old;
2. Spherical equivalent (SE) in either eye: >  − 0.50D and ≤  + 0.75D (based on the average value of automatic refraction under cycloplegia);
3. Astigmatism in either eye: ≤ 1.50D;
4. At least 1 h of near work every day, including non-learning (TV, games, etc.); learning (online learning, punching in); and reading;
5. The uncorrected visual acuity in either eye: ≥ 4.9, and the best corrected visual acuity: ≥ 5.0;
6. Never participated in any clinical trial of myopia control within 3 months or used myopia prevention methods, such as multi-point design defocus glasses, low concentration atropine, repeated low-level red light (RLRL), etc.
7. No manifest strabismus was observed.

Exclusion criteria as follows:

1. Failure to comply with the protocol to obtain reliable study measurements.
2. There is an eye disease that can affect refractive development, such as retinal disease, cataract, and ptosis;
3. The existence of systemic or neurodevelopmental conditions that may affect refractive development;
4. Eye or systemic drugs known to affect the development of myopia or visual acuity through effects on retinal regulation amplitude or intraocular pressure are being used.

### Who will take informed consent? {26a}

Each investigator will personally explain the informed consent form in detail to the subject and their guardian during the recruitment process. This includes providing clear information about the study’s purpose, procedures, potential risks, and benefits, and ensuring sufficient time for them to ask questions and consider their participation. No examinations or interventions will proceed until both the subject and their guardian have provided written informed consent. For children under the age of 8, the guardian will provide consent on their behalf, while verbal assent from the child will be sought whenever appropriate, in accordance with ethical guidelines.

### Additional consent provisions for collection and use of participant data and biological specimens {26b}

N/A: This study does not involve additional data and biospecimens that require subjects’ consent to be collected.

## Interventions

### Explanation for the choice of comparators {6b}

The choice of comparators in this study was guided by the need to evaluate the efficacy of long-term use of DIST and its potential enhancement when combined with the optical defocusing technique in delaying the onset of myopia. This group (daily near work without DIST) serves as the baseline comparator, reflecting typical daily activities that involve prolonged near work without specific interventions. Prolonged near work is a well-documented risk factor for myopia development, but its role in the absence of compensatory measures, such as DIST, remains an important baseline for understanding the natural progression of myopia onset. This control group enables the evaluation of whether DIST alone provides a protective effect against myopia development. By maintaining their habitual near work activities, participants in this group help establish a reference for comparing the effectiveness of interventions.

### Intervention description {11a}

There are two intervention groups in our study: the DIST Group (use of DIST alone) and the Combined Intervention Group (DIST and Optical Defocusing Technique). Participants in these two groups will be asked to use the device during near work, including online learning, playing online games, reading, and similar tasks, with a minimum daily usage of 1 h.

The DIST group is included to investigate whether projecting near scenes into the distance can effectively delay the onset of myopia. This group is critical for isolating the specific effect of DIST as a standalone intervention, independent of other factors. The use of DIST aligns with emerging evidence suggesting that increasing outdoor-like visual experiences or reducing accommodative stress may mitigate myopia progression.

**Fig. 2:**
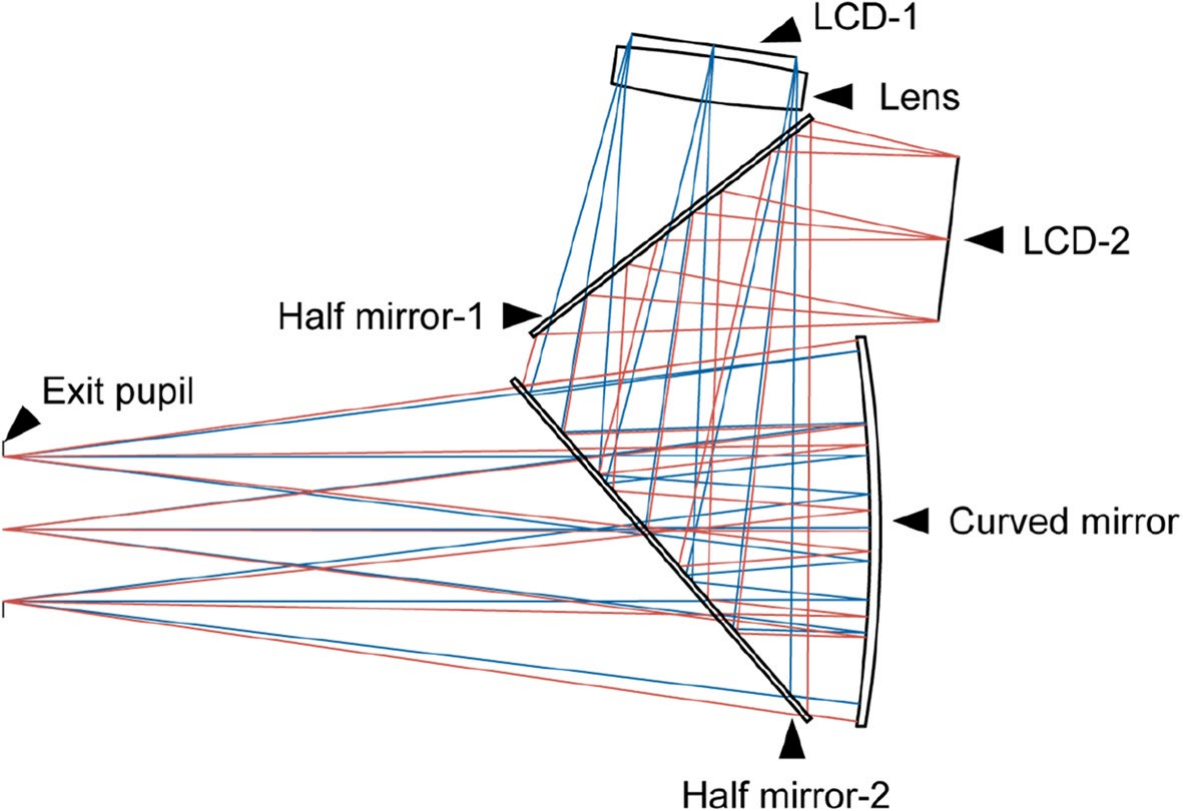
A schematic diagram of the optical path system. The blue lines represent the optical path for distant image display, and the red lines represent the optical path for defocusing intervention

The Combined Intervention group (DIST and Optical Defocusing Technique) evaluates the synergistic effect of combining DIST with an optical defocusing technique. DIST and the optical defocusing technique can be integrated simultaneously through the optical path system (Fig. 2). The system contains two Liquid Crystal Display (LCD) screens, one of which is used to achieve distant-image display, while the other is used to achieve defocus intervention. LCD-1 functions as the object for the distant-image display, while half-mirror-2, the curved mirror, and the lens together form a convex lens optical system. Based on the principles of geometric optics, LCD-1 is positioned within one focal length of this convex lens system. As a result, the light rays entering the eye are divergent, forming an erect, magnified virtual image that enables the perception of a distant image. Similarly, LCD-2 acts as the object for the defocusing pathway, while half-mirror-1, half-mirror-2, and the curved mirror collectively form a convex lens optical system. By positioning LCD-2 between one and two focal lengths of the convex lens optical system, the light rays entering the eye become convergent, thereby achieving defocusing intervention.

Optical path parameters, such as the distance of the distant image, defocus magnitude, display resolution, and luminance at the eye, directly influence the experimental results. In this experiment, the distant image distance in the distant-image display pathway was set to 10 m, the defocus magnitude in the defocusing pathway was set to + 1.0 D, the display resolution was 1080p, and the luminance at the eye was set to approximately 150 nit.

This design enables a comprehensive evaluation of DIST’s efficacy both as a standalone intervention and in combination with optical defocus. By systematically comparing the outcomes across these groups, we aim to identify effective and practical strategies using DIST for delaying myopia onset. This evidence can directly inform clinical recommendations and public health strategies for myopia prevention. We recognize the potential for variability in participants’ adherence to the interventions. To mitigate this, detailed instructions will be provided, and adherence will be monitored through regular follow-ups.

### Criteria for discontinuing or modifying allocated interventions {11b}

Participants may discontinue or have their allocated intervention modified during the trial based on the following criteria:

1. Adverse events or safety concerns. The occurrence of adverse events related to the intervention, such as visual discomfort, eye strain, or other unexpected side effects that compromise the participants’ safety or well-being.
2. Non-compliance with protocol. Persistent non-compliance with the study protocol, including failure to adhere to the prescribed use of DIST or participation in required follow-up visits, despite reasonable efforts to ensure adherence.
3. Participant or guardian decision. Withdrawal of consent by the participant or their guardian for any reason, including personal preferences or perceived lack of benefit from the intervention.
4. Investigator’s discretion. Determination by the investigator that continuing the intervention is not in the participant’s best interest, based on clinical judgment or unforeseen circumstances affecting the study’s integrity.

Participants who discontinue or modify their allocated interventions will remain in the study unless they explicitly request withdrawal. Their data will be included in the Intention-to-Treat analysis to ensure a comprehensive evaluation of the intervention’s efficacy.

### Strategies to improve adherence to interventions {11c}

To enhance participant adherence, each participant will be assigned a dedicated research assistant who will maintain regular contact with the participant’s family through a dedicated WeChat group. The research assistants will offer support by addressing any questions, troubleshooting equipment issues, and sending reminders to ensure timely completion of the intervention and follow-up activities. For experimental groups using DIST, the research team will monitor the device’s usage through backend records once a week to verify adherence to the required usage duration. If any irregular usage patterns are detected, the research assistants will promptly inform the participant’s family and provide personalized guidance to ensure consistent adherence.

### Relevant concomitant care permitted or prohibited during the trial {11d}

Clearly defining permitted and prohibited concomitant care is essential to preserving the integrity of its intervention, protecting participant health and minimising confounding factors.

Permitted concomitant care primarily includes recommendations for healthy visual habits, such as ensuring appropriate reading distance, adequate lighting, and regular outdoor activities. These practices are permitted and encouraged, as they align with standard myopia prevention guidelines.

In contrast, the following are strictly prohibited as concomitant care. First are alternative myopia control interventions. Participants are not allowed to use other myopia control strategies, such as atropine eye drops or other optical interventions (e.g., defocus lenses), during the trial period. Second are experimental therapies. Enrollment in other clinical trials or use of investigational devices or drugs targeting myopia management is prohibited to avoid confounding effects. Last but most important are device modifications. Any unauthorized adjustments or modifications to the DIST device or its usage protocol are also strictly prohibited.

To ensure adherence, all concomitant care received by participants must be reported to the research team and documented during follow-up visits. Any potential impact on study outcomes will be evaluated, and all such data will be recorded in the case report forms (CRFs). Participants and their families will be instructed to notify the study team of any changes in care or new treatments initiated during the trial.

### Provisions for post-trial care {30}

All interventions in our study are considered safe, with no anticipated harm under normal circumstances. However, in the unlikely event that a participant sustains an injury or adverse effect related to the study interventions, the research sponsor will provide comprehensive post-trial care. This includes covering all associated medical expenses and providing appropriate financial compensation based on an assessment conducted by a qualified ophthalmologist.

### Outcomes {12}

#### Primary outcome

##### Proportion of myopia onset

Myopia onset is defined as cycloplegic spherical equivalent refractive error of at least − 0.50D in either eye over 1 year, in accordance with the Refractive Error Study in Children. This outcome assesses the effectiveness of the DIST intervention in delaying the onset of myopia in pre-myopic children.

#### Secondary outcomes

1. *Proportion of fast myopia progressors*: Fast myopia progression is defined as a spherical equivalent myopic shift of at least 0.50 D over 1 year. This outcome helps assess whether the DIST intervention can reduce the incidence of rapid myopic progression.
2. *Changes in spherical equivalent progression (D) at each follow-up point*: This outcome measures the change in spherical equivalent refractive error at each follow-up, serving as a direct indicator of the effect of DIST on refractive progression. It complements the primary outcome by providing additional data on the intervention’s ability to control myopia progression. Ocular parameters of the right eye will be used for analysis to ensure consistency.
3. *Changes in axial length (AL) (mm) at each follow-up point*: AL is a key marker of myopia progression. Monitoring changes in AL will reveal the impact of DIST on ocular growth, which is crucial in myopia development. This outcome provides biological evidence that complements refractive error data, helping to understand how DIST may influence eye structure.

#### Exploratory outcomes

1. *Time from randomization to occurrence of myopia (time-to-event outcome)*: The time from randomization to the occurrence of myopia will be measured as a time-to-event outcome. This endpoint will provide further insights into how quickly DIST delays the onset of myopia compared to the control group.
2. *The association between near-work duration at home and myopia onset*: The duration of near-work activities (e.g., reading, using electronic devices) at home will be correlated with the onset of myopia to investigate environmental factors influencing myopia development. This data will help clarify the role of lifestyle factors in conjunction with DIST intervention.
3. *The association between device usage duration and myopia onset*: The duration of DIST device usage will be correlated with the onset of myopia to explore potential dose–response relationships. This will determine whether longer usage of DIST is associated with a greater effect on myopia prevention, while controlling for other near work behaviors.
4. *Changes in choroidal thickness (ChT) (µm) at 6 and 12 months follow-ups*: Changes in choroidal thickness will be monitored as an exploratory endpoint to assess the potential underlying physiological mechanisms of DIST technology in myopia prevention. Changes in choroidal thickness or blood flow as biomarkers of myopia progression are of significant importance in myopia prevention and control research.
5. *Side effects and adverse events*: The occurrence of side effects and adverse events will be closely monitored throughout the study to ensure the safety of the DIST intervention. This will include any ocular or systemic issues reported by participants, and the severity of these events will be recorded.

### Participant timeline {13}

Screening, baseline, and follow-up time points throughout the study followed a pre-determined schedule, as described in Table 1.

**Table 1:** Timeline of screening, enrollment, and assessments

	Study period				
Procedure	Screening period	Enrolment	Post-assessments		
			3 months (± 14 days)	6 months (± 14 days)	12 months (± 14 days)
Informed consent	X				
Inclusion/Exclusion criteria	X				
General information and relevant medical history	X				
Questionnaire survey		X	X	X	X
Visual acuity(distant)	X		X	X	X
Sub-pupil computer refraction and cornea curvature	X		X	X	X
Sub-pupil subjective refraction	X		X	X	X
Slit-lamp examination	X		X	X	X
Fundus examination	X		X	X	X
Tonometry		X		X	X
Axial length		X	X	X	X
OCT		X		X	X
Paralyze the ciliary muscle		X		X	X
Computer refraction and cornea curvature^a^		X		X	X
Subjective refraction^a^		X		X	X

*OCT* optical coherence tomography

^a^With cycloplegia

### Sample size {14}

To ensure adequate power for detecting differences in myopia incidence between the two intervention groups and the control group, we performed a sample size calculation based on historical data and statistical assumptions. Below, we outline the key parameters and methodological considerations underlying our determination.

#### Outcome measure used for calculations

The primary outcome measure used for this calculation is the incidence of myopia, defined as the proportion of participants developing myopia over 1 year.

#### Test statistic

The test statistic employed is the Pearson chi-square statistic for comparing two independent proportions.

#### Null and alternative hypotheses

##### Null hypothesis (H_0_)

There is no difference in the incidence of myopia between the study arms.

##### Alternative hypothesis (H_1_)

The incidence of myopia in the experimental (intervention) groups is lower than that in the control group.

#### Type I error rate (alpha), power level (e.g., 80% power)

Type I error rate (α): 0.025/2 = 0.0125

Power (1-β): 80%

#### Assumed event rate

Based on a previous retrospective study on the use of low-concentration atropine to prevent myopia onset in pre-myopic children [26], the assumed event rates for a dichotomous outcome are as follows:

- Control group: 54%
- Intervention group: 21%

These rates provide the basis for our assumption that a meaningful reduction in myopia incidence can be observed between groups.

#### Sample size calculation

Using the Pearson chi-square statistic method, we calculated the necessary sample size per group. The software PASS 15.0 and G*Power 3.1 were used to verify the calculations.

Given:

- p₁ (control group event rate) = 0.54
- p₂ (intervention group event rate) = 0.21
- α = 0.0125, β = 0.20
- superiority margin = 0.05

The minimum required sample size per group is approximately 50 participants before adjusting for dropout rates.

#### Adjustment for dropout and attrition

Considering an anticipated dropout rate of 20%, the final required sample size per group is the following:

- Adjusted sample size per group: 64 participants.
- Total sample size for three groups: 192 participants.

#### Method for adjusting calculations for planned interim analyses

At this stage, no adjustments have been made for planned interim analyses. If interim analyses are planned in the study design, the sample size calculation should be adjusted using methods such as the O’Brien–Fleming or Pocock spending functions to control the overall type I error rate.

### Recruitment {15}

To ensure a sufficient sample size, we will implement a two-faceted recruitment strategy targeting schools and paediatric ophthalmology clinics.

1. School-based recruitment
  Wenzhou has a well-established vision screening system, with screenings conducted twice a year (March and September) in all schools. Researchers will collaborate with school health personnel and screening coordinators to identify children in the pre-myopic stage and invite them for further ophthalmic examinations at the hospital.
2. Clinic-based recruitment
  Pediatric ophthalmologists will conduct preliminary screenings in outpatient clinics to identify children who meet the initial eligibility criteria. Parents of eligible children will receive detailed study information, and those expressing interest will be scheduled for a comprehensive baseline assessment to confirm eligibility.

During the recruitment phase, all potential participants and their guardians will receive comprehensive study information, including the research timeline, potential risks, and expected benefits, ensuring fully informed and voluntary participation.

## Assignment of interventions: allocation

### Sequence generation {16a}

A computer-generated randomization schedule will be used to allocate the 192 participants into one control group and two experimental groups in a 1:1:1 ratio. The randomization will be performed using simple randomization without stratification. The sequence will be generated by an independent statistician to ensure allocation concealment and minimize potential bias in the assignment process.

### Concealment mechanism {16b}

A sealed, opaque, and consecutive envelope will be prepared for each participant. Each envelope will contain the group assignment determined by the randomization sequence. The envelopes will be opened sequentially only after a participant is deemed eligible and has provided informed consent, ensuring that the allocation remains concealed until the point of assignment. This approach prevents foreknowledge of group assignments and minimizes selection bias.

### Implementation {16c}

The randomization sequence was generated by the statistical team of the clinical research center of the eye hospital of Wenzhou Medical University. Specialized ophthalmologists in the outpatient clinic will recruit potential participants and conduct baseline assessments. Once participants confirm their enrollment, researchers will assign the corresponding envelope based on the order of participant enrollment. The envelope will then be opened by an independent research assistant, who will use the random number inside the envelope to determine the group assignment.

## Assignment of interventions: blinding

### Who will be blinded {17a}

Due to the nature of the intervention, blinding of participants and intervention providers is not feasible. However, to minimize potential bias, the examination equipment is relatively objective and blinding will be implemented for outcome assessors, data collectors, and data analysts.

Outcome assessors: Independent examiners conducting refraction and AL measurements will be blinded to the participants’ group assignments. They will not be involved in intervention delivery or participant interactions beyond data collection.

Data collectors: Personnel responsible for recording and entering study data will be blinded to group allocation to prevent unconscious bias in data handling.

Data analysts: Statistical analysts will receive a coded dataset without group identifiers, ensuring that data analysis remains objective.

Blinding procedures will be strictly maintained throughout the study to enhance the reliability and validity of the findings.

### Procedure for unblinding if needed {17b}

N/A.

## Data collection and management

### Plans for assessment and collection of outcomes {18a}

At baseline and during each follow-up, researchers will provide paper-based questionnaires for guardians to complete, and the results will be entered into a spreadsheet for analysis. All other clinical assessments will be performed by experienced clinicians using relevant measuring instruments (as specified in item 12). Measurements will be repeated to ensure the reliability and validity of the data collection. Meanwhile, patients’ general information, medical history, baseline and follow-up results, adverse event reports, and trial summaries will be recorded in the CRFs by researchers on time. All researchers and clinicians will undergo training on participant recruitment, assessment, intervention, and data backup before participating in the study.

Cycloplegic autorefraction will be performed using the Topcon KR800 autorefractor unit following the cycloplegia regimen of 1% cyclopentolate (Cyclogyl, AlconConvreur). The procedure involves administering two drops of 1% cyclopentolate separated by a 5-min interval, followed by a 30-min resting period after the final instillation. Complete cycloplegia is confirmed by the absence of fluctuation in retinoscopy findings under cycloplegic conditions in children or by performing autorefraction combined with careful subjective refraction. Spherical equivalent is calculated as spherical power plus half of the cylinder power. Cycloplegic measurements will be performed at baseline, the 6-month follow-up, and the 12-month follow-up to assess the primary outcome.

Ocular AL will be measured on a Zeiss IOL Master 700 (Carl Zeiss Meditec Inc.) using noncontact partial coherence interferometry. Perform six measurements of total AL, anterior chamber length, and corneal curvatures. At least three measurements are identical or almost identical. The ChT will be measured using Wevis OCT (Wevis Imaging Technology Co., Ltd.) in the Angio 6 × 6 512 R4 mode. Each image covers a 6 × 6 mm area centered on the fovea. Researchers will use the OCT’s built-in software to automatically center the image and segment the choroidal thickness. Choroidal thickness is defined as the perpendicular distance between the outer choroid-sclera margin and the retinal pigment epithelium–Bruch’s membrane complex. The subfields analyzed are delineated using the standard ETDRS 9-segment grid, including the central zone (0–1 mm), the four quadrants of the inner ring (1–3 mm), and the four quadrants of the outer ring (3–6 mm). After measurement, all images will be checked for signal quality and segmentation accuracy. If the signal quality is too low or segmentation errors are present, the measurement will be repeated.

Questionnaires are used to assess children’s subjective visual experience during near work. The assessment focuses on visual comfort (e.g., fatigue, eye pain, blurred vision) and behavioral manifestations such as lack of concentration or reading difficulties during near tasks. Additionally, evaluating the time spent on outdoor activities and near work provides insights into myopia risk associated with lifestyle habits.

### Plans to promote participant retention and complete follow-up {18b}

To ensure high participant retention and complete follow-up throughout the study, a comprehensive strategy will be implemented. This strategy will focus on maintaining participant engagement, minimizing dropout rates, and addressing potential challenges that may arise during the study.

1. Engagement and retention strategies

- Regular communication: Participants will be regularly contacted via phone calls, text messages, or mobile applications to provide reminders of upcoming appointments and to maintain engagement.
- Flexible scheduling: Follow-up visits will be scheduled at times convenient for the participants and their families, such as weekends and holidays.
- Incentives: To further encourage retention, participants will receive small incentives, such as gift cards or study-related materials, upon completion of key milestones (e.g., baseline, 6-month, and 12-month visits).

2. Handling withdrawals or treatment modifications

In the event that a participant decides to withdraw or is unable to complete the study, their outcome data up to the point of withdrawal will be collected. This ensures that even if they do not complete the entire study, the data they have contributed remains valuable.

- Reasons for withdrawal: Participants who withdraw will be asked to provide the reason for their decision (e.g., personal, logistical, or health-related issues), which will be documented for further analysis and to inform potential improvements in future studies.
- Changes in treatment plan: If participants need to switch to an alternative treatment, the research team will collect relevant outcome data before the change is made, ensuring that the participant’s previous data remains part of the analysis. This will allow the study to account for the impact of treatment changes on the outcomes.

3. Follow-up and data collection

In the event of missing data or uncompleted follow-up visits, efforts will be made to contact participants to reschedule and collect the necessary data. If a participant cannot be reached for any follow-up visit, the last available data will be included in the analysis, and the participant will be considered as missing for subsequent follow-up points.

By employing these strategies, we aim to minimize participant dropout, ensure high-quality follow-up data, and maximize the retention of participants throughout the 1-year study period.

### Data management {19}

Effective data management is critical for ensuring the integrity, security, and reliability of the data collected throughout the study. A comprehensive plan will be implemented for data entry, coding, confidentiality, and storage, with quality assurance measures to ensure accurate and complete data collection.

- Case report form: Case report forms, which will be in paper form, will be used to record clinical data in the trial, and case report forms. As original material, the CRFs will not be changed at will. The relevant information of all patients participating in the trial will be recorded timely and truthfully.
- Data entry: All data collected will be entered into an electronic data capture (WMU-EDC (Wenzhou Medical University, Wenzhou, Zhejiang, China) database. To prevent errors, double data entry will be used, where two separate team members will independently enter the data, and discrepancies will be resolved through a reconciliation process.
- Data quality control measures

Range checks: To identify potential errors, automated range checks will be applied to the data during entry. For instance, measurements outside of predefined acceptable ranges (e.g., refraction values) will trigger a prompt for review.

Consistency checks: Periodic consistency checks will be conducted to detect any discrepancies or outliers in the dataset. These checks will compare newly entered data with historical data or other clinical parameters.

Training for data collectors: All staff involved in data collection and entry will undergo standardized training on the study protocols, measurement techniques, and data entry processes to minimize human error and improve consistency.

Data security: Data will be stored on secure cloud servers with encrypted access. Hard copies of any records will be kept in locked file cabinets, accessible only to authorized personnel. An audit trail will be maintained for all data-related actions, including data entry, modifications, and access, ensuring full traceability and accountability.

Data access and monitoring: Only authorized research staff will have access to the data, and data access permissions will be strictly controlled and documented. Regular internal audits will be conducted to ensure that data collection and management processes are being followed correctly. Data integrity will be routinely monitored, and any potential issues will be addressed promptly.

By implementing these measures, we aim to ensure data accuracy, security, and confidentiality while maintaining the highest standards of data quality throughout the study.

### Confidentiality {27}

To ensure the confidentiality of participants’ personal information throughout the study, a series of measures will be implemented to protect privacy during the pre-study phase, throughout the study, and after completion. These measures will govern the collection, sharing, and storage of personal data for both potential and enrolled participants.

1. Participant informed consent
  All participants will sign an informed consent form before entering the study, which will clearly explain how their personal information will be collected, used, stored, and shared. Participants will have the right to withdraw consent at any time and request the destruction of their personal data. The research team will ensure that participants fully understand how their personal data will be protected, and they will be given sufficient time and opportunity to ask questions or express concerns.
2. Data collection and storage
  During the study, all personal information related to participants will be stored using coding systems to ensure that identifying information is separate from study data. Each participant will be assigned a unique identification number, and only this number will be used to identify them.
  Personal identifying information (e.g., name, phone number, address) will be kept in separate files from the study data and will be accessible only to authorized personnel.
  Participant personal information will be stored in encrypted storage systems (e.g., cloud storage) with regular security updates and checks to maintain data protection.
3. Data sharing and access
  Only authorized researchers and relevant personnel will have access to participants’ personal information, and this access will be granted strictly for research purposes. All access to data will be controlled through a permission-based system, ensuring that only necessary personnel can view sensitive information.
  Personal information will not be shared unless legally required or for specific research needs. When reporting results to external entities, all published data will be anonymized to prevent the disclosure of any personal identifiers.
4. Post-study data management
  After the study concludes, all personal information related to participants will continue to be handled according to confidentiality agreements and will only be used for purposes directly related to the study (e.g., data analysis, final reporting).
  Original data will be retained for a specified period and then securely destroyed to ensure that no personal information is accessible or disclosed after the study’s conclusion.
  If the data needs to be used for subsequent research or publications after the study has ended, participants will be re-consented, and appropriate confidentiality measures will be applied.
  Through these measures, we will ensure the protection of participants’ privacy and comply with all relevant legal and ethical standards to safeguard the confidentiality of data and participants’ rights.

### Plans for collection, laboratory evaluation and storage of biological specimens for genetic or molecular analysis in this trial/future use {33}

N/A: As indicated in the outcome measures (item 12), our study does not involve the collection of biological samples.

## Statistical methods

### Statistical methods for primary and secondary outcomes {20a}

1. Analysis of primary outcome
  In this participant-based study, the primary outcome is the proportion of myopia onset. This outcome is measured as a binary (nominal) variable. Participants are classified as either having experienced the onset of myopia (Yes) or not (No), based on whether their cycloplegic spherical equivalent refractive error reaches or exceeds − 0.50 D in either eye at the 1-year follow-up. This is a single endpoint measure, assessed once at the end of the 1-year follow-up period. It provides a proportion of participants who experience the onset of myopia. To test the effectiveness of DIST on the primary outcomes, logistic regression including trend analysis will be used, which can both examine the overall relationship and detect potential trends related to different treatments. In addition, covariates such as baseline spherical equivalent refractive error, age, gender, and study group (Intervention vs. Control) will be included to ensure a parsimonious logistic regression model while controlling for factors known to influence myopia onset and progression.
  The primary analysis will be conducted on the ITT population, which is defined as all randomized participants with at least one post-baseline measurement. For all analyses, the underlying model assumptions will be rigorously evaluated. For instance, when applying logistic regression to assess the binary primary outcome, we will verify the linearity of the logit for continuous covariates using methods such as the Box–Tidwell test, assess multicollinearity via variance inflation factors (VIF), and examine residuals and influence statistics (e.g., Cook’s distance) to identify any influential outliers. Missing data will be addressed using multiple imputations by chained equations under the assumption of data missing at random (MAR), with sensitivity analyses planned to assess the impact of outliers, nonadherence, and loss to follow-up.
2. Analysis of secondary outcomes
  The secondary outcomes are intended to capture aspects of the intervention effect that are not fully reflected by the primary outcome. For example, even if the primary outcome (myopia onset) does not show a statistically significant difference, significant changes in spherical equivalent or axial length may still be observed, thereby indicating a potential benefit of the intervention in slowing myopia progression.
  For the three secondary outcomes: (1) the proportion of fast progressors over 1 year, (2) changes in spherical equivalent progression, and (3) changes in AL (mm) at each follow-up point, the following statistical procedures will be employed:
  1. Proportion of fast progressors (binary outcome)
    This endpoint will be analyzed using logistic regression. The model will estimate the odds of being classified as a fast progressor over 1 year, comparing the intervention group to the control group. Covariates to be included in the model are baseline spherical equivalent refractive error (continuous), age (continuous), and gender (categorical), as these factors have been shown in prior research to influence myopia progression. The study group (intervention vs. control) will be the primary factor of interest. These covariates are selected based on their theoretical relevance and empirical support, ensuring a parsimonious model that controls for potential confounding without overfitting.
  2. Changes in spherical equivalent progression (continuous outcome)
    The change in spherical equivalent progression over time will be analyzed using a repeated measures mixed-effects model. This model will account for within-subject correlations across multiple follow-up points and will include fixed effects for the treatment group, time (as a categorical or continuous variable depending on the observed pattern), and the interaction between treatment and time. Baseline spherical equivalent refractive error, age, and gender will be included as covariates to adjust for initial differences and known predictors of refractive change. The rationale for these covariates is their established association with myopia progression, ensuring that the model is both explanatory and parsimonious. Model selection procedures, such as stepwise backward elimination based on the Akaike Information Criterion (AIC) or likelihood ratio tests, will be applied to refine the final model.
  3. Changes in AL (mm)
    Similar to the spherical equivalent endpoint, changes in axial length (measured in mm) at each follow-up will be analyzed using a repeated measures mixed-effects model or an analysis of covariance (ANCOVA) for longitudinal data. The model will include fixed effects for treatment, time, and their interaction. Covariates will include baseline axial length, baseline spherical equivalent, age, and gender. These factors are chosen because they are known to influence ocular growth and, consequently, axial elongation. As with the other endpoints, a parsimonious model will be achieved by using stepwise selection methods or likelihood ratio tests to retain only covariates that contribute significantly to explaining the variation in axial length change.
    The results of these analyses will be presented as adjusted odds ratios with 95% confidence intervals for the binary outcome and as least-squares means (LSMEANS) with standard errors for the continuous outcomes. In addition, p-values will be reported to assess the statistical significance of the observed differences between intervention groups.
3. Analysis of exploratory outcomes
  1. Time-to-myopia onset analysis
    For comparing the time to myopia onset (time-to-event outcome) among 3 treatment groups, we all create a number-at-risk table and use the Kaplan–Meier method to estimate survival curves, followed by the log-rank test to assess differences statistically.
  2. Myopia onset associations
    A logistic regression model will be built with myopia onset as the dependent variable and near-work duration, device usage duration, and confounding factors as independent variables. The near-work duration was derived from the near-work log cards and questionnaires, with the daily averages taken for analysis. Odds ratio (OR) and its 95% confidence interval will be estimated to determine associations while controlling for confounding.
  3. Dose–response relationship analysis
    We will group near work and device usage durations (e.g., by quartiles). Use the Cochrance–Armitage trend test to analyze if there’s a dose–response relationship. Curve fitting (such as polynomial regression) can also be used to further explore the non-linear relationship between them.
  4. Choroidal thickness changes
    For exploring changes in choroidal thickness during different follow-up periods among three groups, repeated measures analysis of variance (ANOVA) will be used. If significant effects are found, post hoc tests (like Bonferroni or Tukey’s) will determine specific differences.

### Interim analyses {21b}

N/A

### Methods for additional analyses (e.g., subgroup analyses) {20b}

Subgroup analysis will be conducted based on the total duration of near work and the usage duration of devices in this study. The aim is to explore the impacts of these two factors on AL growth and the incidence rate of myopia among the subjects.

Firstly, we will divide the subjects into different subgroups according to specific criteria regarding the durations of near work and device usage.

Then, appropriate statistical methods will be employed for the analysis. To analyze the impact on the AL growth, which is a continuous variable, we will consider using analysis of variance (ANOVA) or regression analysis to compare the differences in AL changes among different subgroups and to determine whether there are significant associations between the durations of near work/device usage and the AL growth. In this process, relevant covariates such as age, gender, baseline refractive status, and outdoor activity time will be adjusted to control for potential confounding effects.

For the analysis of the impact on the incidence rate of myopia, which is a binary variable, logistic regression will be utilized. The myopia incidence will be set as the dependent variable, while the durations of near work and device usage will be the main independent variables. Additionally, the same set of covariates as mentioned above will be included in the model to account for confounding factors and to obtain more accurate estimates of the odds ratios and their corresponding confidence intervals, which can reflect the strength and significance of the associations between these factors and the myopia incidence.

Furthermore, interaction terms between the durations of near work and device usage might be considered in the models to explore whether there are combined effects of these two factors on the outcomes of interest. Through this comprehensive subgroup analysis, we expect to gain a more detailed and in-depth understanding of how the durations of near work and device usage influence the AL growth and the myopia incidence among the subjects.

### Subgroup analysis of secondary outcomes

Subgroup analyses will be conducted to investigate two primary factors among pre-myopic children: (1) the total duration of device usage, and (2) the duration of near work. The objective is to determine how these factors affect key myopia prevention indicators—including the primary endpoint (proportion of myopia onset) and secondary outcomes (proportion of fast progressors, change in spherical equivalent progression, and change in axial length).

### Stratification and definition

Participants will be stratified into subgroups based on predetermined thresholds for both device usage and near work duration. For the purposes of this study, we will operationally define high-intensity near work behavior as a daily at-home near work duration of 2 h or more, while near work behavior lasting less than 2 h per day will be classified as low-intensity.

All participants will be included in the analysis. Adherence, defined as the ratio of actual device usage duration to planned usage duration, is calculated as both daily (daily adherence (%) = actual daily training duration/planned daily training duration × 100%) and overall (overall adherence (%) = ∑ (actual daily training duration)/∑ (planned daily training duration) × 100%). Subsequently, according to the adherence categories established by the Pediatric Eye Disease Investigator Group (PEDIG) [27, 28] (“excellent” (76%–100%), “good” (51%–75%), “fair” (26%–50%), and “poor” (0%–25%)), participants with an average daily device usage of 30 min or more will be classified as having high adherence, while those with less than 30 min per day will be classified as low adherence. These thresholds may be refined based on the statistical distribution of the relevant data at the end of the study.

### Statistical procedures

#### Continuous outcomes (axial length, spherical equivalent change)

For outcomes measured on a continuous scale (i.e., axial length growth and change in spherical equivalent), we will use analysis of variance (ANOVA) or linear regression models. In these models, the subgroup classifications (based on device usage and near work duration) will be the main independent variables. Adjustments will be made for covariates such as age, gender, baseline refractive error, and outdoor activity time to control for potential confounding. Model selection will follow a strategy to achieve parsimony—incorporating only those covariates that are theoretically justified and empirically significant.

#### Binary outcomes (myopia incidence and proportion of fast progressors)

For binary outcomes, logistic regression will be utilized. Here, the dependent variable will be the incidence of myopia (or classification as a fast progressor), and the primary predictors will be the categorized durations of device usage and near work. The same set of covariates (age, gender, baseline refractive error, outdoor activity time) will be included to adjust for confounding. Odds ratios with corresponding 95% confidence intervals will be calculated to quantify the associations.

#### Interaction analysis

Interaction terms between device usage duration and near work duration will be incorporated into the regression models to explore potential synergistic effects on myopia prevention outcomes.

#### Rationale and interpretation

The independent analysis of these subgroups is designed to provide a deeper understanding of how both device usage and near work independently and jointly influence myopia progression indicators. This analysis is not dependent on the primary endpoint results; rather, it is intended to complement the overall findings and help refine targeted intervention strategies for high-risk pre-myopic children.

The results will be presented as adjusted means (for continuous outcomes) and adjusted odds ratios (for binary outcomes) along with their standard errors or 95% confidence intervals. This comprehensive subgroup analysis will yield insights that may inform future recommendations for reducing near work-related visual stress and optimizing the use of the DIST device in myopia prevention.

### Methods in analysis to handle protocol non-adherence and any statistical methods to handle missing data {20c}

To comprehensively assess the impact of the devices on delaying myopia onset, we will conduct data analyses using both the Intention-to-Treat (ITT) analysis as the primary result and the Per-Protocol (PP) analysis as the secondary result, along with appropriate handling of missing data in both processes.

#### ITT analysis (primary result)

The ITT analysis will be carried out to reflect the real-world application effect of the devices in preventing myopia onset. In case a participant drops out or has missing data during the follow-up period, we will use the Last Observation Carried Forward (LOCF) method. This ensures that all randomly assigned participants are retained in the analysis, maintaining the integrity of the randomization and providing a comprehensive view of the overall effect of the devices in a real-life scenario.

#### PP analysis (secondary result)

The PP analysis will be employed as a secondary result to focus on the effect among those participants who strictly adhered to the use of the devices and followed the study protocol precisely. Only those subjects who completed all the required procedures, used the devices as instructed (e.g., for the specified duration and frequency), and attended all the follow-up visits without any major deviations from the protocol will be included in this analysis. To maintain the purity of the analysis, we will use direct exclusion to handle the missing data.

In conclusion, by utilizing both ITT and PP analyses with appropriate handling of missing data, we expect to obtain a comprehensive and in-depth understanding of the effect of the DIST on delaying myopia onset in pre-myopic children from different perspectives, which will contribute to a more robust and reliable evaluation of the efficacy of these devices in this context.

### Plans to give access to the full protocol, participant-level data, and statistical code {31c}

In our clinical research, we have well-defined plans for sharing important study materials to ensure transparency and reproducibility.

Firstly, the full protocol of our study will be published in the Trials. Additionally, when the research is concluded and the corresponding article is published, the full protocol will also be attached as Supplementary materials at the end of the main text.

Secondly, regarding the raw data, we will make it available on the data platform of our hospital’s clinical research center. However, this will take place after the official publication of the research article. To access the raw data, interested parties are required to fill out a corresponding application form. They can then submit the application to the corresponding author for review. During this process, strict ethical and privacy safeguards are in place. The raw data will be anonymized to protect the identities of the participants by removing or encrypting personal identifiers such as names, addresses, and social security numbers. This ensures that while enabling the sharing of valuable data for further research and verification purposes, the privacy rights of the participants are duly respected.

## Oversight and monitoring

### Composition of the coordinating center and trial steering committee {5d}

In this single-center clinical trial, the coordinating center is the hospital’s Clinical Research Center. The trial steering committee plays a key role, composed of 2 hospital senior ophthalmology experts, 3 statistics experts (1 external), the project leader, and the sponsor’s leader. It is responsible for determining the full trial protocol, reviewing progress, and setting endpoints.

The Ethics Committee safeguards ethical aspects. The Endpoint Adjudication Committee, with 2 experts, ensures endpoint accuracy. And the Data Management Team, having 2 admins and 2 quality control staff, maintains data quality.

The project research team consists of clinical ophthalmologists, laboratory researchers, and research assistants from the sponsor. They jointly handle tasks like participant recruitment, intervention implementation, outcome evaluation, data management, and analysis. Each task has an experienced leader. The steering committee oversees the whole study, while the research team holds monthly meetings to discuss progress and solve trial-related issues. Together, these components work in harmony to support the trial’s success and ensure its scientific integrity and high quality.

### Composition of the data monitoring committee, its role and reporting structure {21a}

The Data Monitoring Committee (DMC) of the Clinical Trial Center at the Eye Hospital, Wenzhou Medical University, will be responsible for data monitoring and overseeing the entire study. The committee members are independent and do not include any personnel involved in this study, ensuring no conflicts of interest. All raw data will be stored with the Data Monitoring Committee and will not be made publicly available before the results are published.

### Adverse event reporting and harms {22}

In our study, a meticulous approach is adopted for adverse event reporting and addressing potential harms.

We utilize paper-based CRFs to record relevant information. During each follow-up visit, investigators will actively inquire about participants’ ocular discomfort manifestations, such as visual fatigue, dry eyes, and foreign body sensation. Meanwhile, we also collect information on other unexpected reactions like having a cold or a fever, including details about the duration of these symptoms as well as the methods used for relief and cure.

Based on the participants’ reported reactions, investigators will carefully assess the correlation between these events and the trial. Appropriate handling plans will then be formulated accordingly.

Moreover, participants are encouraged to contact our investigators immediately if they experience any ocular discomfort symptoms or unexpected reactions during the use of relevant items in the study. Our investigators will provide timely and correct handling solutions to ensure the well-being of the participants and minimize potential harms. Through such a comprehensive process, we strive to closely monitor and effectively manage adverse events and safeguard the health and safety of all participants involved in our study.

### Frequency and plans for auditing trial conduct {23}

The trial steering committee regularly reviews the progress of the study (such as progress monitoring, decision modification, et al.) at intervals of every 3 months. It plays a crucial and pivotal role in our project. It is independent from both the searchers and the sponsors.

### Plans for communicating important protocol amendments to relevant parties (e.g., trial participants, ethical committees) {25}

Before the commencement of the study, specifically during the design stage, the research protocol needs to undergo multiple rounds of demonstration and modification before it can be implemented. During the implementation process, if there are contents that truly require adjustments, for example, regarding the age in the inclusion criteria of this study, which was originally set as 6–12 years old and has been changed to 6–10 years old, the hospital’s Clinical Research Center has a clear process in place to determine whether such modifications will affect the scientific integrity, ethical considerations, and practical implementation of the study. This involves relevant parties such as the investigators, CRC, who participate in the trial and the trial registration institutions.

### Dissemination plans {31a}

The researchers and sponsors have developed a comprehensive plan for disseminating the trial results to various audiences, including trial participants, healthcare practitioners, the general public, and other relevant groups.

Firstly, the trial results may be shared at academic conferences and events. After we complete the whole trial and data analysis, the complete results of the trial will be published in scientific journals. Secondly, after publication, all raw data and statistical results will be made publicly available on the Chinese Clinical Trial Registry website. However, this data sharing will be carried out in compliance with ethical and legal requirements, ensuring that participant privacy is protected. Lastly, it should be noted that there may be certain publication restrictions. For example, if the study involves intellectual property rights or confidential agreements with collaborating partners, the dissemination of specific details may be limited until the relevant permissions are obtained. In such cases, we will ensure transparency by clearly communicating the reasons for these restrictions to the interested parties.

## Discussion

The rising global prevalence of myopia has made its prevention and control a critical public health challenge, particularly among children and adolescents [29–32]. While managing myopia progression is important, preventing its onset is an even more valuable strategy. The International Myopia Institute (IMI) highlights the concept of “pre-myopia,” which refers to a refractive state of ≤  + 0.75 D and >  − 0.50 D [33]. In this state, combined with baseline refractive error, age, and other quantifiable risk factors, the likelihood of developing myopia in the future is significantly high, warranting preventive intervention. Although some preventive measures are available, including optical interventions, time spent outdoors, and behavioural influences [23, 34], the limitations of these methods underscore the need for innovative myopia prevention strategies. This study focuses on addressing the issue of near work in children, aiming to evaluate the effectiveness of DIST as a supplementary approach for pre-myopia interventions.

Given that the participants are still pre-myopic children, we rationally include a control group that performed conventional near work to investigate whether DIST alone was effective in delaying the onset of myopia on the one hand and whether it would be better when combined with optical defocusing techniques on the other. The questionnaire is designed to capture critical factors influencing insufficient hyperopic reserve at this stage, such as parental myopia, the participants’ eye habits, and time spent in outdoor activities. We also introduced a near-work log card to record additional near-work duration at home, as near work at school is currently unavoidable. This allows us to evaluate the potential impact of DIST during discretionary near-work periods. In addition, due to the lack of previous studies using similar equipment as a reference, the sample size calculation in our study may result in insufficient statistical power, potentially affecting the reliability of the results. However, our results could provide a foundation for future research.

In the current educational situation, increasing academic pressure on children and adolescents has made it challenging to ensure sufficient outdoor activities and exposure to natural light. Compared to other devices that can reduce near work, such as projectors or large-screen televisions, DIST requires only a small space and offers a more seamless integration with children’s near-task activities, allowing their eyes to relax by focusing on distant objects without interfering with their learning tasks, thereby reducing visual strain. If the effectiveness of DIST is demonstrated, it could serve as a promising supplementary strategy for myopia prevention. Moreover, if DIST proves to be more effective when combined with widely recognized optical defocusing techniques, it will open up opportunities for further individualization of optical defocus parameters to cater to patient-specific needs, unlocking greater clinical potential. Although we measured changes in choroidal thickness in an attempt to explore the underlying mechanisms, this study primarily discusses the clinical applicability of the DIST intervention; further investigation and refinement are needed to fully elucidate the mechanisms.

## Trial status

Version and date of this protocol: version 2, March 30, 2026.

Recruitment schedule: started in March 2024 and completed in September 2025.

## Data Availability

Due to ethical restrictions, we cannot share the full dataset collected. However, the datasets used and analyzed in this study are available upon request from the corresponding author.

## Abbreviations

DIST: Distant-Image Screen Technology
SE: Spherical equivalent
RLRL: Repeated low-level red light
CRFs: Case report forms
AL: Axis length
ChT: Choroidal thickness
OCT: Optical coherence tomography
ITT: Intent-to-treat
PP: Per-protocol
VIF: Variance inflation factors
MAR: Missing at random
AIC: Akaike Information Criterion
ANCOVA: Analysis of covariance
LSMEANS: Least-squares means
OR: Odds ratio
ANOVA: Analysis of variance
LOCF: Last observation carried forward
DMC: Data Monitoring Committee
IMI: International Myopia Institute

## References

[1] Holden BA, Fricke TR, Wilson DA, Jong M, Naidoo KS, Sankaridurg P, et al. Global prevalence of myopia and high myopia and temporal trends from 2000 through 2050. Ophthalmology. 2016;123(5):1036–42. 10.1016/j.ophtha.2016.01.006. 26875007 10.1016/j.ophtha.2016.01.006

[2] Modjtahedi BS, Abbott RL, Fong DS, Lum F, Tan D, Ang M, et al. Reducing the global burden of myopia by delaying the onset of myopia and reducing myopic progression in children. Ophthalmology. 2021;128(6):816–26. 10.1016/j.ophtha.2020.10.040. 33388160 10.1016/j.ophtha.2020.10.040

[3] You QS, Wu LJ, Duan JL, Luo YX, Liu LJ, Li X, et al. Factors associated with myopia in school children in China: the Beijing childhood eye study. PLoS ONE. 2012;7(12):e52668. 10.1371/journal.pone.0052668. 23300738 10.1371/journal.pone.0052668 PMC3531363

[4] Risk factors for idiopathic rhegmatogenous retinal detachment. The Eye Disease Case-Control Study Group. Am J Epidemiol. 1993;137(7):749–57 PubMed PMID: 8484366. 8484366

[5] Liang YB, Friedman DS, Wong TY, Zhan SY, Sun LP, Wang JJ, et al. Prevalence and causes of low vision and blindness in a rural chinese adult population: the Handan Eye Study. Ophthalmology. 2008;115(11):1965–72. 10.1016/j.ophtha.2008.05.030. PubMed PMID: 18684506. 18684506 10.1016/j.ophtha.2008.05.030

[6] Kanthan GL, Mitchell P, Rochtchina E, Cumming RG, Wang JJ. Myopia and the long-term incidence of cataract and cataract surgery: the Blue Mountains Eye Study. Clin Exp Ophthalmol. 2014;42(4):347–53. 10.1111/ceo.12206. 24024555 10.1111/ceo.12206

[7] Mitchell P, Hourihan F, Sandbach J, Wang JJ. The relationship between glaucoma and myopia: the Blue Mountains Eye Study. Ophthalmology. 1999;106(10):2010–5. 10.1016/s0161-6420(99)90416-5. 10519600 10.1016/s0161-6420(99)90416-5

[8] Holy C, Kulkarni K, Brennan NA. Predicting costs and disability from the myopia epidemic–a worldwide economic and social model. Invest Ophthalmol Vis Sci. 2019;60(9):5466–5466.

[9] Sankaridurg P, Tahhan N, Kandel H, Naduvilath T, Zou H, Frick KD, et al. IMI impact of myopia. Invest Ophthalmol Vis Sci. 2021;62(5):2. 10.1167/iovs.62.5.2. 33909036 10.1167/iovs.62.5.2 PMC8083082

[10] Zhang W, Chang S, Jiang J, Yu M, Chen S, Hu Y, et al. Association between vision-related quality of life and mental health status in myopia children using various optical correction aids. Contact Lens Anterior Eye. 2024;47(5):102287. 10.1016/j.clae.2024.102287. 39191536 10.1016/j.clae.2024.102287

[11] Goldstand S, Koslowe KC, Parush S. Vision, visual-information processing, and academic performance among seventh-grade schoolchildren: a more significant relationship than we thought? Am J Occup Ther. 2005;59(4):377–89. 10.5014/ajot.59.4.377. 16124204 10.5014/ajot.59.4.377

[12] Dudovitz RN, Izadpanah N, Chung PJ, Slusser W. Parent, teacher, and student perspectives on how corrective lenses improve child wellbeing and school function. Matern Child Health J. 2016;20(5):974–83. 10.1007/s10995-015-1882-z. 26649878 10.1007/s10995-015-1882-z PMC4826825

[13] Enthoven CA, Tideman JWL, Polling JR, Yang-Huang J, Raat H, Klaver CCW. The impact of computer use on myopia development in childhood: the Generation R study. Prev Med. 2020;132:105988. 10.1016/j.ypmed.2020.105988. 31954142 10.1016/j.ypmed.2020.105988

[14] Biswas S, El Kareh A, Qureshi M, Lee DMX, Sun CH, Lam JSH, et al. The influence of the environment and lifestyle on myopia. J Physiol Anthropol. 2024;43(1):7. 10.1186/s40101-024-00354-7. 38297353 10.1186/s40101-024-00354-7 PMC10829372

[15] Sivaraman V, Rizwana JH, Ramani K, Price H, Calver R, Pardhan S, et al. Near work-induced transient myopia in Indian subjects. Clin Exp Optom. 2015;98(6):541–6. 10.1111/cxo.12306. 26497844 10.1111/cxo.12306

[16] Huang HM, Chang DST, Wu PC. The association between near work activities and myopia in children-a systematic review and meta-analysis. PLoS ONE. 2015;10(10):e0140419. 10.1371/journal.pone.0140419. 26485393 10.1371/journal.pone.0140419 PMC4618477

[17] Eppenberger LS, Grzybowski A, Schmetterer L, Ang M. Myopia control: are we ready for an evidence based approach? Ophthalmol Ther. 2024;13(6):1453–77. 10.1007/s40123-024-00951-w. 38710983 10.1007/s40123-024-00951-w PMC11109072

[18] Liang X, Wei S, Zhao S, Li SM, An W, Sun Y, et al. Investigation of choroidal blood flow and thickness changes induced by near work in young adults. Curr Eye Res. 2023;48(10):939–48. 10.1080/02713683.2023.2222234. 37303164 10.1080/02713683.2023.2222234

[19] Chhabra S, Rathi M, Sachdeva S, Rustagi IM, Soni D, Dhania S. Association of near work and dim light with myopia among 1400 school children in a district in North India. Indian J Ophthalmol. 2022;70(9):3369–72. 10.4103/ijo.IJO_634_22. 36018123 10.4103/ijo.IJO_634_22 PMC9675519

[20] Zhen Y, Zhang W, Shen J, Cheng D, Shen W, Wang NL. The clinical value of using a distant-image screen for reading and learning. Chin J Ophthalmol. 2022;58(12):1045–50. 10.3760/cma.j.cn112142-20220106-00004. 10.3760/cma.j.cn112142-20220106-00004 36480886

[21] Anstice NS, Phillips JR. Effect of dual-focus soft contact lens wear on axial myopia progression in children. Ophthalmology. 2011;118(6):1152–61. 10.1016/j.ophtha.2010.10.035. 21276616 10.1016/j.ophtha.2010.10.035

[22] Berntsen DA, Barr CD, Mutti DO, Zadnik K. Peripheral defocus and myopia progression in myopic children randomly assigned to wear single vision and progressive addition lenses. Invest Ophthalmol Vis Sci. 2013;54(8):5761–70. 10.1167/iovs.13-11904. 23838771 10.1167/iovs.13-11904 PMC3755539

[23] Wildsoet CF, Chia A, Cho P, Guggenheim JA, Polling JR, Read S, et al. IMI-interventions myopia institute: interventions for controlling myopia onset and progression report. Invest Ophthalmol Vis Sci. 2019;60(3):M106–31. 10.1167/iovs.18-25958. PubMed PMID: 30817829. 30817829 10.1167/iovs.18-25958

[24] Cho P, Cheung SW. Retardation of myopia in orthokeratology (ROMIO) study: a 2-year randomized clinical trial. Invest Ophthalmol Vis Sci. 2012;53(11):7077–85. 10.1167/iovs.12-10565. 22969068 10.1167/iovs.12-10565

[25] Chan AW, Tetzlaff JM, Gotzsche PC, Altman DG, Mann H, Berlin JA, et al. SPIRIT 2013 explanation and elaboration: guidance for protocols of clinical trials. BMJ. 2013;346(jan08 15):e7586. 10.1136/bmj.e7586. 23303884 10.1136/bmj.e7586 PMC3541470

[26] Fang PC, Chung MY, Yu HJ, Wu PC. Prevention of myopia onset with 0.025% atropine in premyopic children. J Ocul Pharmacol Ther. 2010;26(4):341–5. 10.1089/jop.2009.0135. 20698798 10.1089/jop.2009.0135

[27] Brin TA, Chow A, Carter C, Oremus M, Bobier W, Thompson B. Efficacy of vision-based treatments for children and teens with amblyopia: a systematic review and meta-analysis of randomised controlled trials. BMJ Open Ophthalmol. 2021;6(1):e000657. 10.1136/bmjophth-2020-000657. 33912684 10.1136/bmjophth-2020-000657 PMC8043000

[28] The Pediatric Eye Disease Investigator Group. A randomized trial of atropine vs patching for treatment of moderate amblyopia in children. Arch Ophthalmol. 2002;120(3):268–78. 10.1001/archopht.120.3.268. 11879129 10.1001/archopht.120.3.268

[29] Dong L, Kang YK, Li Y, Wei WB, Jonas JB. Prevalence and time trends of myopia in children and adolescents in China: a systemic review and meta-analysis. Retina. 2020;40(3):399–411. 10.1097/IAE.0000000000002590. 31259808 10.1097/IAE.0000000000002590

[30] Ding BY, Shih YF, Lin LLK, Hsiao CK, Wang IJ. Myopia among schoolchildren in East Asia and Singapore. Surv Ophthalmol. 2017;62(5):677–97. 10.1016/j.survophthal.2017.03.006. 28359704 10.1016/j.survophthal.2017.03.006

[31] Yotsukura E, Torii H, Inokuchi M, Tokumura M, Uchino M, Nakamura K, et al. Current prevalence of myopia and association of myopia with environmental factors among schoolchildren in Japan. JAMA Ophthalmol. 2019;137(11):1233–9. 10.1001/jamaophthalmol.2019.3103. 31415060 10.1001/jamaophthalmol.2019.3103 PMC6696729

[32] Xiang F, He M, Zeng Y, Mai J, Rose KA, Morgan IG. Increases in the prevalence of reduced visual acuity and myopia in Chinese children in Guangzhou over the past 20 years. Eye Lond. 2013;27(12):1353–8. 10.1038/eye.2013.194. 24008929 10.1038/eye.2013.194 PMC3869515

[33] Flitcroft DI, He M, Jonas JB, Jong M, Naidoo K, Ohno-Matsui K, et al. IMI-defining and classifying myopia: a proposed set of standards for clinical and epidemiologic studies. Invest Ophthalmol Vis Sci. 2019;60(3):M20-30. 10.1167/iovs.18-25957. 30817826 10.1167/iovs.18-25957 PMC6735818

[34] Jonas JB, Ang M, Cho P, Guggenheim JA, He MG, Jong M, et al. IMI prevention of myopia and its progression. Invest Ophthalmol Vis Sci. 2021;62(5):6. 10.1167/iovs.62.5.6. 33909032 10.1167/iovs.62.5.6 PMC8083117

